# A new blood parasite of the accentor birds: description, molecular characterization, phylogenetic relationships and distribution

**DOI:** 10.1017/S0031182024000878

**Published:** 2024-09

**Authors:** Gediminas Valkiūnas, Tatjana A. Iezhova, Mélanie Duc, Jenny C. Dunn, Staffan Bensch

**Affiliations:** 1 P. B. Šivickis Laboratory of Parasitology, Nature Research Centre, Vilnius, Lithuania; 2 School of Life Sciences, Huxley Building, Keele University, Newcastle-under-Lyme, Staffordshire, UK; 3 Joseph Banks Laboratories, School of Life and Environmental Sciences, University of Lincoln, Lincoln, UK; 4 School of Biology, University of Leeds, Leeds, UK; 5 Department of Biology, Lund University, Lund, Sweden

**Keywords:** full mitochondrial genome, geographical and host distribution, *Haemoproteus bobricklefsi* sp. nov, Haemosporidian parasites, phylogenetic relationships

## Abstract

*Haemoproteus bobricklefsi* sp. nov. (Haemosporida, Haemoproteidae) was found in the dunnock *Prunella modularis* and represents the first blood parasite described in accentor birds of the Prunellidae. The description is based on the morphology of blood stages and includes information about a barcoding segment of the mitochondrial cytochrome *b* gene (lineage hDUNNO01) and the full mitochondrial genome, which can be used for identification and diagnosis of this infection. The new parasite can be readily distinguished from described species of haemoproteids parasitizing passeriform birds due to markedly variable position of nuclei in advanced and fully grown macrogametocytes. Illustrations of blood stages of the new species are given, and phylogenetic analyses based on partial mitochondrial cytochrome *b* gene sequences and the full mitochondrial genome identified the closely related lineages. DNA haplotype networks showed that transmission occurs in Europe and North America. This parasite was found in the dunnock in Europe and several species of the Passerellidae in North America. It is probably of Holarctic distribution, with the highest reported prevalence in the UK. The parasite distribution seems to be geographically patchy, with preference for areas of relatively cool climates. Phylogenetic analysis suggests that *H. bobricklefsi* sp. nov. belongs to the *Parahaemoproteus* subgenus and is probably transmitted by biting midges belonging to *Culicoides* (Ceratopogonidae). The available data on molecular occurrence indicate that this pathogen is prone to abortive development, so worth attention in regard of consequences for bird health.

## Graphical abstract

**Figure:**
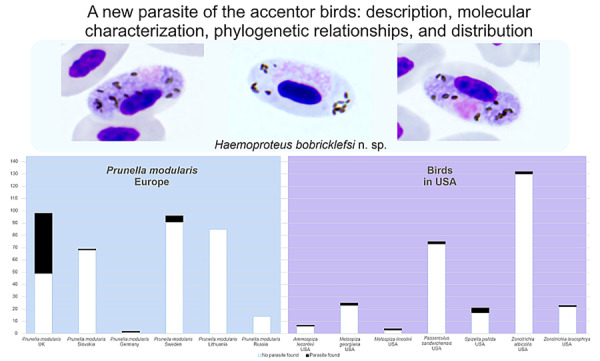

## Introduction

*Haemoproteus* species (Haemosporida, Haemoproteidae) are cosmopolitan and diverse blood parasites that are flourishing in birds (Atkinson, 2008; Braga *et al*., 2011; Clark *et al*., 2014; Santiago-Alarcon and Marzal, 2020). They are obligate heteroxenous protists, with the sporogony in blood-sucking insects of the Ceratopogonidae and Hippoboscidae and the asexual multiplication (merogony) and gametocyte production in avian hosts. *Haemoproteus* parasites are important for bird health due to disease (haemoproteosis), which can be accompanied by severe tissue pathologies (Atkinson *et al*., 1986; Atkinson, 2008; Valkiūnas and Iezhova, 2017; Kelly *et al*., 2018; Yoshimoto *et al*., 2021; Duc *et al*., 2023; Himmel *et al*., 2024). They can also be virulent and even lethal in blood-sucking insects due to the damage caused by migrating ookinetes during high infections, but this issue remains insufficiently investigated in wildlife (Valkiūnas *et al*., 2014; Bukauskaitė *et al*., 2016).

The application of polymerase chain reaction (PCR)-based and microscopic approaches in parallel is the gold standard in haemosporidian diversity and ecology studies (Goulding *et al*., 2016; Mantilla *et al*., 2016; Chagas *et al*., 2018; Duc *et al*., 2023). Over 2000 mitochondrial cytochrome b (*cytb*) lineages have been identified and serve as convenient markers for *Haemoproteus* species diagnostics (Bensch *et al*., 2009; MalAvi database http://130.235.244.92/Malavi, accessed in February 2024). In all, 178 morphospecies of these parasites have been described (Valkiūnas and Iezhova, 2022; Duc *et al*., 2023). However, most of the genetic lineages remain non-identified to species levels and the majority of described morphospecies remain uncharacterized molecularly. This is unfortunate for disease diagnostics, particularly because of difficulties in detection of recently recognized abortive haemosporidian infections, which are common in wildlife and can cause avian disorders (Valkiūnas, 2015; Valkiūnas and Iezhova, 2017; Ortiz-Catedral *et al*., 2019). Such infections occur when the invasive stages of haemosporidians (gametocytes or sporozoites) appear in the wrong hosts. In such non-susceptible or only party susceptible animals (blood-sucking insects or birds), they manage to develop only partially but cannot complete the life cycle and produce the corresponding invasive stages, thus being a dead end for transmission. These infections can be virulent and even lethal in birds but remain poorly studied because they are difficult to diagnose by microscopic examination due to the absence of gametocytes in blood (Valkiūnas, 2015; Ortiz-Catedral *et al*., 2019). PCR-based diagnostics can detect abortive haemosporidian infections (Moens *et al*., 2016) due to the presence of DNA templates in the circulation that might be the remnants of tissue stages in birds (Palinauskas *et al*., 2016), or ookinetes in vectors (Valkiūnas *et al*., 2013, 2014). PCR-based diagnostics help to recognize host individuals with abortive haemosporidian infections for further targeting of histological and molecular examination.

It is worth mentioning that reports only based on genetic lineages in avian hosts and vectors provide incomplete information about transmission at a study site. Such reports confirm the presence of parasite DNA templates for PCR amplifications but cannot prove whether the infected animals are the competent hosts, in which the maturation of invasive stages occurs. The latter is important for infection spread. In other words, solely PCR-based information about the prevalence of infection should be treated with caution in epidemiology and ecology research. Microscopic examination allows the visualization of the haemosporidian invasive stages and can complement the sensitive PCR-based observations. The application of these 2 approaches in parallel provides good results in haemosporidian diversity and ecology research (Goulding *et al*., 2016; Chagas *et al*., 2018; Bensch and Hellgren, 2020; Duc *et al*., 2023). However, morphological parasite description remains a slow process, which is much behind the rapid accumulation of DNA sequence information. The identification of molecular markers for haemosporidian species identification and the linkage of this information with taxonomic parasite descriptions are timely tasks for current molecular and parasitology research.

Taxonomic studies on *Haemoproteus* species remain challenging, particularly due to the following obstacles. First, haemosporidian taxonomy at the species level is based mainly on morphological features of blood stages, but since parasitaemia is predominantly low in naturally infected animals, it is hard to find samples that enable the visualization of all blood stages, which are necessary for species identification. This requires extensive sampling of hosts as well as time-consuming microscopic examination of blood films. The latter also needs taxonomic training. Additionally, the identification of haemosporidian species in sporogony stages (Chagas *et al*., 2018) and tissue stages (Duc *et al*., 2023; Himmel *et al*., 2024) remains insufficiently developed using morphological characters. In other words, the limited available information for these life cycle stages can currently hardly help in practical taxonomic research; further accumulation of information is necessary. Second, co-infections of parasites belonging to same genus or subgenus predominate in wildlife and are obstacles for distinguishing morphologically similar species of the same genera, particularly by inexperienced taxonomists. Despite these difficulties, taxonomic research on avian haemosporidian parasites is experiencing an acceleration due to opportunities provided by the application of molecular characterization (barcoding) methods, which are used in parallel with microscopic examination of blood samples (Valkiūnas and Iezhova, 2022). The use of genetic information provides opportunities to group together the visualized blood stages of the same lineage from different host individuals and thus to use even low parasitaemia samples for identification and description of morphospecies. Importantly, the availability of barcoding DNA sequences makes morphological taxonomic research readily repeatable. Meanwhile, *Haemoproteus* parasites remain unidentified or non-characterized molecularly in birds of many families. For example, *Haemoproteus cytb* lineages were found in birds of the Aegithalidae, Certhidae, Prunellidae, Regulidae, Troglodytidae – common European birds – but information about the parasites' species identity is absent. In the tropics the situation is even worse, and haemoproteids remain undescribed in birds of most families (Santiago-Alarcon and Marzal, 2020). The lack of linkages between the taxonomic and the rapidly accumulating molecular information slows down research on the biology of avian haemosporidian parasites and limits our understanding of the consequences of haemosporidiosis in wildlife.

This study aimed to describe and characterize molecularly the first species of *Haemoproteus* (Haemoproteidae, Haemosporida) in accentor birds (Passeriformes, Prunellidae, *Prunella*) – an endemic genus to the Palearctic region consisting of 13 species (Liu *et al*., 2017) – as well as contributing information about some patterns of distribution of this infection.

## Materials and methods

### Study sites and sampling

The material was collected at 4 study sites in Europe (Table 1). In all, 261 individual dunnocks *Prunella modularis* were caught mainly by mist nets. Blood was collected by puncturing the brachial vein, and the blood films were prepared on ready-to-use glass slides immediately after withdrawal; they were air-dried, fixed in absolute methanol and stained with Giemsa using a standard protocol (Valkiūnas, 2005). For all birds sampled after 2004, approximately 35 *μ*L of blood were either collected in heparinized micro capillaries and fixed in SET-buffer (Hellgren *et al*., 2004) or collected in non-heparinized capillary tubes and frozen directly; the samples were maintained at +4°C in the field and −20°C in the laboratory prior to molecular analysis.

**Table 1.** Prevalence of *Haemoproteus bobricklefsi* sp. nov. (lineage hDUNNO01) in various parts of the Holarctic

Continent, bird family and species ^a^	Country	Location	Coordinates	Years	Prevalence, % (found/screened)	Reference
*Europe*						
Prunellidae						
*Prunella modularis*	UK*	Lincoln	53°12′N, 0°32′W	2017–2019	50 (49/98)^b^^,^^c^	Woodrow *et al*. (2023), not published
	Slovakia	Drienovec	48°36′N, 020°54′E	2017–2019	1.4 (1/69)^b^	Sujanová *et al*. (2021)
	Germany	Marburg	50°50′N, 008°40′E	2019–2022	50 (1/2)^b^	Strehmann *et al*. (2023)
	Sweden*	Krankesjön	55°41′N, 13°26′E	2013–2017	5.2 (5/96)^b^^,^^c^	Ellis *et al*. (2020)
	Lithuania*	Ventė Cape	55°20′ N, 21°11′E	2015–2022	0 (0/82)^d^	Not published
	Russia*	Curonian Spit	55°5ʹ N, 20°44′ E	1982, 1983, 1994, 1997, 2003, 2004, 2012, 2018, 2019	0 (0/14)^d^	Valkiūnas (1985), not published
Overall prevalence					21.5 (56/261)	
*North America*						
Passerellidae						
*Ammospiza leconteii*	USA	Minnesota	47°57′N, 096°27′W	1999–2019	16.7 (1/6)^b^	Fecchio *et al*. (2023), MalAvi database
*Melospiza georgiana*	USA	Pennsylvania	39°58′N, 075°29′W	1999–2019	8.7 (2/23)^b^	Fecchio *et al*. (2023), MalAvi database
*M. lincolnii*	USA	Pennsylvania	39°58′N, 075°29′W	1999–2019	33.3 (1/3)^b^	Fecchio *et al*. (2023), MalAvi database
*Passerculus sandwichensis*	USA	Minnesota	47°57′N, 096°27′W	1999–2019	2.7 (2/73)^b^	Fecchio *et al*. (2023), MalAvi database
*Spizella pallida*	USA	Minnesota	47°57′N, 096°27′W	1999–2019	23.5 (4/17)^b^	Fecchio *et al*. (2023), MalAvi database
*Zonotrichia albicollis*	USA	Pennsylvania	39°58′N, 075°29′W	1999–2019	1.5 (2/130)^b^	Fecchio *et al*. (2023), MalAvi database
*Z. leucophrys*	USA	Alaska	63°19′N, 148°13′W	Not available	4.5 (1/22)^b^	Galen *et al*. (2018)
Overall prevalence					4.7 (13/274)	

Sampling sites of this study were marked with asterisks (*).

a The lineage hDUNNO01 has also been reported from 6 species in Alaska, however information about the prevalence was absent. These are the common redpoll *Acanthis flammea* (Fringillidae), fox sparrow *Passerella iliaca* (Passerellidae), dark-eyed junco *Junco hyemalis* (Passerellidae), varied thrush *Ixoreus naevius* (Turdidae), white-crowned sparrow *Zonotrichia leucophrys* (Passerellidae) and willow ptarmigan *Lagopus lagopus* (Phasianidae) in 2011–2012 (Oakgrove *et al*., 2014). This lineage was found in yellowhammer *Emberiza citrinella* (Emberizidae), common blackbird *Turdus merula* (Turdidae) and willow warbler *Phylloscopus trochilus* (Phylloscopidae) in the UK (Dunn *et al*., 2014; Woodrow *et al*., 2023; J. C. D., not published).

b PCR-based diagnostics.

c Partial microscopic examination of samples, which were PCR-positive. Gametocytes were seen in all PCR-positive samples screened by microscopy.

d Microscopic examination of all samples.

### Microscopic examination and parasite morphology

Samples tested by microscopic examination are shown in Table 1. Several models of light microscopes were used for examination of blood films according to the standard protocol (Valkiūnas, 2005). The Olympus BX61 microscope equipped with an Olympus DP70 digital camera and the imaging software AnalySIS FIVE (Olympus, Tokyo, Japan) were used for preparation of parasite illustrations and measurements.

### Deoxyribonucleic acid extraction, PCR and sequencing

DNA was extracted following the ammonium acetate protocol (Sambrook and Russel, 2001) or using DNeasy blood and tissue kits (Qiagen, Manchester, UK) following the manufacturer's instructions. DNA concentrations were measured, and each sample was diluted to a concentration of 25 ng *μ*L^−1^.

Samples from the UK were screened using 3 primer sets, each with the same forward primer (UNIVF 5′-CAYATAYTAAGAGAAYTATGGAG-3′) and a different reverse primer (UNIVR1: 5′-GCATTATATCWGGATGWGNTAATGG-3′; UNIVR2: 5′-ARAGGAGTARCATATCTATCWAC-3′; UNIVR3: 5′-ATAGAAAGMYAAGAAATACCATTC-3′) (Drovetski *et al*., 2014). Reactions were carried out in 10 *μ*L reaction volumes containing 5 *μ*L QIAGEN 2X Multiplex PCR buffer (Qiagen), 0.2 *μ*L each primer (10 mm), 3.6 *μ*L RNase-free water and 1 *μ*L template DNA. Each PCR run contained a sample from a bird with known infection, and a sample containing water in place of DNA, to ensure successful PCR amplification and lack of contamination, respectively. The PCR protocol involved denaturation at 95°C for 15 min followed by 42 cycles of denaturation at 94°C for 30 s, annealing for 30 s at primer-specific temperatures (UNIVR1: 54°C; UNIVR2: 52°C; UNIVR3: 53°C) and 45 s extension at 72°C, followed by a final terminal extension at 72°C for 10 min. PCR protocols were carried out using a BioRad T100 Thermal Cycler (BioRad, Hercules, CA, USA). PCR products were visualized on a 1% agarose gel stained with GelRed (Cambridge Bioscience, Cambridge, UK), and all positive samples were sent for bidirectional sequencing by Macrogen Europe (Amsterdam, the Netherlands).

Samples from Sweden were screened during a previous study (Ellis *et al*., 2020) using a nested PCR with 25 *μ*L *reaction* volumes (Taq DNA Polymerase [Sigma-Aldrich, Stockholm, Sweden]) and following original protocols (Bensch *et al*., 2000; Hellgren *et al*., 2004). PCR products were visualized on 2% agarose gel stained with GelRed, and all positive samples were sent for bidirectional Sanger sequencing (Lund University, Sweden).

Three individuals positive by nested PCR and microscopic examination were further used for nested long-range PCR to amplify the whole mitochondrial genome of the parasite, following the protocol established by Ciloglu *et al*. (2020), with modification of the annealing temperatures. Briefly, the first PCR started with 98°C for 30 s, followed by 20 cycles of denaturation at 94°C for 10 s, annealing at 53°C for 30 s and elongation at 68°C for 10 min, followed by an extension of 10 min at 68°C, with the primers mtDNA-F (5′-GAGGATTCTCTCCACACTTCAATTCGTACTTC-3′) and mtDNA-R (5′-CAGGAAAATWATAGACCGAACCTTGGACTC-3′) (Pacheco *et al*., 2018). The second PCR differed by having 30 cycles, an annealing step at 55°C for 20 s and the elongation step for 7 min, with the primers mtDNA-F inner (5′-CACTTCAATTCGTACTTCCACTACCA-3′) and mtDNA-R inner (5′-GACCGAACCTTGGACTCTTGA-3′) (Ciloglu *et al*., 2020). PCR products were visualized on 2% agarose gels after electrophoresis at 90 V for 90 min. Two of the 3 tested samples were positive and were purified, quantified, sheared and prepared for sequencing on an Illumina MiSeq Instrument (University of Lund, Sweden) following the methods by Ciloglu *et al*. (2020).

The obtained sequences were analysed using the Geneious Prime 2022.1.1 software (https://www.geneious.com). Sanger sequences were trimmed, aligned and checked for possible co-infections (double peaks in chromatograms). Lineages were identified by BLASTing consensus sequences in GenBank (the US National Centre for Biotechnology Information) and MalAvi (http://130.235.244.92/Malavi/index.html) databases.

Illumina sequences were analysed following the protocol by Ciloglu *et al*. (2020).

Sequences were deposited in GenBank (Sanger sequence: PP358260, Illumina sequences PP557255, PP557256).

### Phylogenetic analysis

To determine the phylogenetic relationships of *H. bobricklefsi* sp. nov. (hDUNNO01) with closely related *Haemoproteus* spp. lineages, a Bayesian inference tree was calculated with the second best-fit model, GTR + I + G (on criteria AIC, AICc, BIC), based on jmodeltest2.1 (Guindon and Gascuel, 2003; Darriba *et al*., 2012) using sequences of 478 bp. The best-fit model was TIM2 + I + G, which is not a model implemented in MrBayes, but which can be substituted by GTR + I + G model (Lecocq *et al*., 2013). In total, 44 *Haemoproteus* spp. sequences were used, for which the species identity has been described and the samples collected from the type hosts, with pSYAT05 *Plasmodium vaughani* (KY653792) as the outgroup. Mr. Bayes plugin v3.2.6 (Huelsenbeck and Ronquist, 2001) was run in Geneious, for 5 million generations, sampled every 200th generation, and discarding the first 25% trees as ‘burn-in’ for the consensus tree.

Full mitochondrial genome sequences deposited in GenBank from previous studies (Matta *et al*., 2014; Mantilla *et al*., 2016; Pacheco *et al*., 2018; Ciloglu *et al*., 2020; Musa, 2023) and the newly sequenced hDUNNO01 were used to calculate a second phylogeny. The best-fit model, GTC + I + G (on AIC, AICc, BIC and DT criteria) from jmodeltest2.1 (Guindon and Gascuel, 2003; Darriba *et al*., 2012), was run in Geneious using MrBayes plugin with the same parameters as the above-described phylogeny.

Genetic distances between lineages were calculated in Geneious and extracted from the corresponding matrix (100 minus percentage identity matrix).

### DNA haplotype network of cytb lineages closely related to *Haemoproteus bobricklefsi* sp. nov. (hDUNNO01)

To visualize the geographic and host distribution of *H. bobricklefsi* sp. nov. (hDUNNO01), a DNA haplotype network was constructed based on the 478 bp *cytb* barcode sequences and information from the MalAvi database. Lineages for which sequences were within 1.5% difference from the sequence of hDUNNO01 were selected for the haplotype network. This cut-off includes all lineages directly connected to DUNNO01 in the network and those connecting to its closest neighbours. The Geneious' alignment of the sequences was used to calculate a Median-Joining Network using the software PopArt (Bandelt *et al*., 1999; Leigh and Bryant, 2015) with default settings. Trait information (geographical sampling location or family of the sampled host) was extracted from MalAvi database (‘Host and Sites’ table) and related to the geographical areas determined by the United Nations geoscheme (https://unstats.un.org/unsd/methodology/m49/, accessed in January 2024), or to the families of the host species (checked on https://ebird.org, accessed in February 2024). Graphics were finalized in PopArt and CorelDraw 2019 (RRID:SDR_01435, https://www.coreldraw.com/en/).

### Statistical analysis

Student's *t-*test for independent samples was used to determine statistically significant differences between mean linear parameters of gametocytes and their host cells. Prevalences of infection were compared by Yates corrected *χ*^2^ test. A *P* value of 0.05 or less was considered significant. The statistical analyses were carried out using the ‘Statistica 7’ package.

## Results

### Prevalence of *H. bobricklefsi* sp. nov. (hDUNNO01) in dunnocks

Based on well-represented samples of dunnocks (*n* ⩾ 14), the prevalence of the lineage hDUNNO01 was low (< 6%) at all study sites, except in the UK where this parasite was detected in 50% of dunnocks (Table 1). Microscopic examination did not reveal *H. bobricklefsi* sp. nov. from the 2 sites located around the Curonian Lagoon (Lithuania and Russia), indicating that this infection is absent or rare. The significantly higher prevalence of hDUNNO01 in the UK in comparison to other examined sites in Europe (for all sites *χ*^2^ > 5, *P* < 0.05) suggests the existence of favourable transmission conditions in the UK, but not so good in more eastern sites of this bird's range in Europe. Intensity of parasitaemia was low (⩽ 0.1%) in all microscopy-positive samples. In dunnocks (*n* = 17, slides examined in the UK), mean parasitaemia was 0.79% (range 0.09–4.77%). All PCR-positive dunnocks that were examined by microscope in the UK (*n* = 17) had gametocytes in the blood.

### Phylogenetic analysis

**Figure 1.**
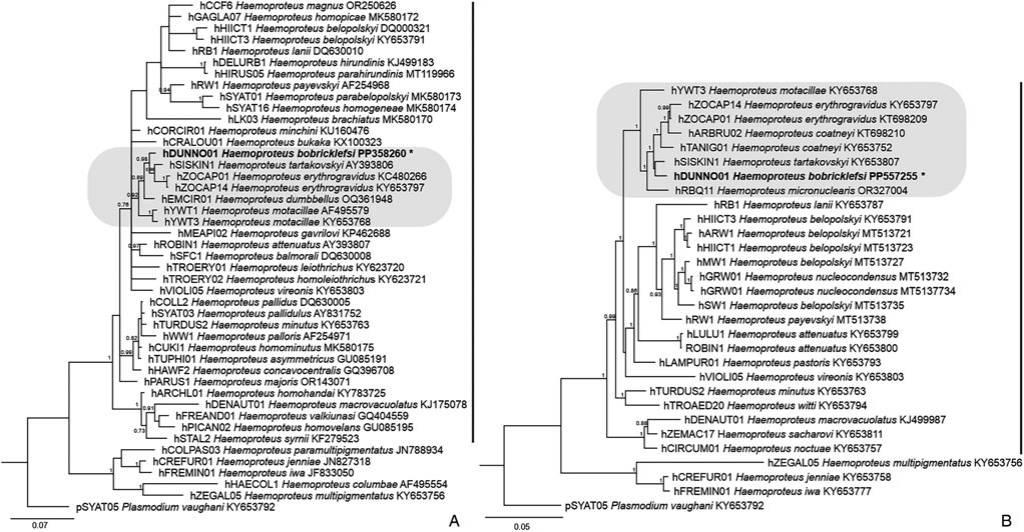
Bayesian phylogenetic analysis of the partial cytochrome *b* gene sequences which were linked to the morphospecies (A) and of the full mitochondrial genome (B) of haemosporidian parasites. In all, 44 sequences (A) and 29 sequences (B) of *Haemoproteus* species were used in the phylogenies, with the lineage pSYAT05 of *Plasmodium vaughani* as outgroup. *Haemoproteus bobricklefsi* sp. nov. (lineage hDUNNO01) clusters with species of the *Parahaemoproteus* subgenus. MalAvi lineage codes, parasite species names and GenBank accession numbers were indicated. Posterior probabilities greater than 0.7 were provided. Vertical bars indicate lineages of parasites belonging to *Parahaemoproteus* subgenus. Grey boxes indicate lineages, which are closely related to *H. bobricklefsi* sp. nov.

The phylogenies based on the 478 bp of the *cytb* gene and the full mtDNA placed the lineage hDUNNO01 of *H. bobricklefsi* sp. nov. within a clade of parasites belonging to subgenus *Parahaemoproteus* (Fig. 1). The closest described species were *H. tartakovskyi* (lineage hSISKIN1), *H. erythrogravidus* (hZOCAP01, hZOCAP14) and *H. motacillae* (hYWT3). More polytomies were observed in the phylogeny based on the partial *cytb* gene, compared to the whole mtDNA phylogeny, in which most of the nodes have high support. Genetic differences in partial *cytb* and the full mtDNA between the lineage hDUNNO01 and the above-mentioned lineages were as follows: hSISKIN1 – 1.88 and 2.18%, hZOCAP01 – 2.51 and 2.55%, hZOCAP14 – 2.3 and 2.79%; hYWT3 – 3.35 and 3.17%.

### Parasite description

*Haemoproteus (Parahaemoproteus) bobricklefsi* sp. nov. (lineage hDUNNO01)

*Type host:* Dunnock *Prunella modularis* (Linnaeus, 1758) (Passeriformes, Prunellidae).

*DNA sequences:* Mitochondrial *cytb* lineage hDUNNO01 (479 bp, GenBank accession no. PP358260).

*Additional hosts:* The lineage hDUNNO01 was recorded in 16 bird species, mostly of the Passerellidae (Table 1). However, it remains to be established in which of these avian species the parasite can complete its life cycle and develop gametocytes.

*Type locality:* UK, Potterhanworth, Lincolnshire, UK. 53°12′N 0°32′W.

*Site of infection:* Mature erythrocytes, no other data.

*Prevalence:* Markedly varied in different sites in Europe (Table 1), with a tendency to decrease from west to the east area of dunnock distribution.

*Vectors:* Unknown; probably *Culicoides* biting midges, as the phylogenetic analysis suggests (see Discussion).

*Type specimens:* Hapantotype (accession no. 49706 NS, parasitaemia intensity is ~0.1%, *P. modularis*, 7 June 2019, Lincolnshire, UK, collected by J. C. Dunn) was deposited in Nature Research Centre (NRC), Vilnius, Lithuania. Parahapantotype preparations were from the same bird species; they were deposited at NRC (49707 NS, 49708 NS, 22 April 2021 and 49709 NS, 22 February 2023, Lincolnshire, UK, collected by J. C. Dunn in the UK, and 49711 NS, 14 June 2014, Krankesjön, Sweden, collected by S. Bensch in Sweden) and Queensland Museum, Queensland, Australia (G466303, 17 April 2023 collected by J. C. Dunn in Lincolnshire, UK and G466304, 23 August 2017, Krankesjön, Sweden, collected by S. Bensch in Sweden).

*Additional material:* Voucher blood films from dunnocks (accession nos. 49713–49722 NS) were deposited in NRC, Vilnius, Lithuania.

*Etymology:* This species was named in honour of Professor Robert E. Ricklefs, University of Missouri-St. Louis, USA, in recognition of his contributions to the studies of ecology and evolutionary biology of haemosporidian parasites.

*ZooBank registration:* urn:lsid:zoobank.org:pub:46424228-4849-41F3-AE9C-44D3514ED5A3.

**Figure 2.**
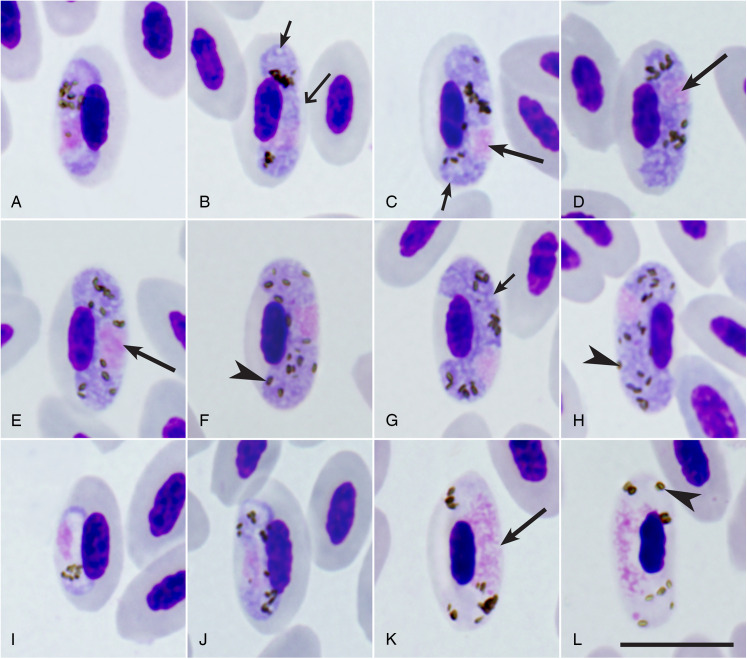
Gametocytes of *Haemoproteus bobricklefsi* sp. nov. (lineage hDUNNO01) from the blood of its type host, the dunnock *Prunella modularis*: a, b, i, j – young gametocytes, c–h – macrogametocytes, k, l – microgametocytes. Note that both subterminal (c, d, g, h) and central (e, f) positions of nuclei occur in macrogametocytes, a characteristic feature of this species. Dumbbell-shaped gametocytes occur among the growing parasites (b, j) but not among the fully grown ones (f–h, k, l). Long simple arrows – nuclei of gametocytes. Short simple arrows – vacuoles. Triangle wide long arrow – a space between gametocyte and envelope of infected erythrocyte. Simple arrowheads – pigment granules. Giemsa-stained blood film. Scale bar = 10 *μ*m. All images are from the hapantotype preparation.

*Young gametocytes (Fig. 2a):* The earliest gametocytes seen anywhere in infected erythrocytes. As gametocytes grow, they adhere to the nuclei of erythrocytes, extend longitudinally along the nuclei, but do not displace or only slightly displace them laterally (Fig. 2a,b,i,j). Few small pigment granules were visible; they were usually grouped. Gametocyte outline is even or slightly wavy.

*Macrogametocytes (Fig. 2b*–*h*, Table 2*):* The cytoplasm is homogenous in appearance, contains small vacuoles; volutin granules not seen. Outline is even or slightly amoeboid. Growing gametocytes are closely appressed to nuclei of infected erythrocytes; their ends closely adhere to the host cell envelope, however the central part of the pellicle often does not extend to the erythrocyte envelope, causing a ‘dip’ and giving a dumbbell-like appearance (Fig. 2b). Advanced (Fig. 2c–e) and fully grown gametocytes (Fig. 2g and h) are closely appressed to the nuclei and envelop of infected erythrocytes; they fill up poles of erythrocytes (Fig. 2g and h) and slightly displace the nuclei laterally (Fig. 2c–h). Circumnuclear or close to circumnuclear gametocytes not seen. The parasite nuclei are big, of vague outlines, variable in form and position. The unstable position of nuclei in macrogametocytes is a characteristic feature of this species: the subterminal or close to subterminal position of nuclei (Fig. 2g and h) predominates (75%), but the strictly central position (Fig. 2e) is also common (25%). Pigment granules are of medium size (0.5–1 *μ*m), mostly elongate or oval, but sometimes roundish; they are randomly scattered in the cytoplasm. Mean linear dimensions of infected erythrocytes are only slightly increased in comparison to uninfected erythrocytes (Table 2). Mean area of infected erythrocytes is increased slightly (*P* < 0.05).

**Table 2.** Morphometry of host cells and mature gametocytes of *Haemoproteus bobricklefsi* n. sp. from the blood of *Prunella modularis*

Feature	Measurements (*μ*m)^a^
Uninfected erythrocyte	
Length	11.6–13.4 (12.4 ± 0.5)
Width	5.1–6.6 (6.1 ± 0.4)
Area	54.6–67.4 (61.8 ± 3.7)
Uninfected erythrocyte nucleus	
Length	5.0–6.9 (5.8 ± 0.6)
Width	1.9–3.1 (2.4 ± 0.3)
Area	8.7–14.2 (10.8 ± 1.4)
*Macrogametocyte*	
Infected erythrocyte	
Length	11.7–14.3 (13.2 ± 0.6)
Width	6.1–7.3 (6.6 ± 0.3)
Area	62.9–78.3 (70.0 ± 4.8)
Infected erythrocyte nucleus	
Length	5.2–6.3 (5.8 ± 0.3)
Width	1.8–2.6 (2.2 ± 0.2)
Area	9.9–12.7 (11.0 ± 0.8)
Gametocyte	
Length	13.3–16.8 (14.8 ± 0.9)
Width	2.5–3.5 (2.9 ± 0.3)
Area	38.5–53.0 (46.9 ± 3.9)
Gametocyte nucleus	
Length	2.7–4.1 (3.3 ± 0.4)
Width	2.1–3.3 (2.7 ± 0.3)
Area	5.1–8.9 (7.4 ± 1.1)
Pigment granules#	11.0–16.0 (14.4 ± 1.6)
NDR^b^	0.5–0.8 (0.7 ± 0.1)
*Microgametocyte*	
Infected erythrocyte	
Length	12.3−15.1 (13.9 ± 0.7)
Width	5.3–6.9 (6.2 ± 0.4)
Area	58.9–81.3 (70.6 ± 5.0)
Infected erythrocyte nucleus	
Length	5.0–6.4 (5.6 ± 0.3)
Width	2.0–2.8 (2.3 ± 0.2)
Area	8.9–15.2 (10.9 ± 1.4)
Gametocyte	
Length	16.2–20.8 (18.7 ± 1.3)
Width	2.3–3.0 (2.5 ± 0.2)
Area	43.2–56.8 (51.0 ± 4.2)
Gametocyte nucleus	
Length	7.9–12.8 (9.9 ± 1.4)
Width	1.6–3.0 (2.4 ± 0.3)
Area	12.6–24.9 (19.8 ± 3.5)
Pigment granules	10.0–15.0 (12.0 ± 1.4)
NDR^b^	0.5–0.8 (0.7 ± 0.1)

a All measurements (*n* = 21) are given in micrometres. Minimum and maximum values are provided, followed in parentheses by the arithmetic mean and standard deviation.

b NDR = nucleus displacement ratio according to Bennett and Campbell (1972).

*Microgametocytes (Fig. 2j*–*l):* The general configuration is as for macrogametocytes with the usual haemosporidian sexual dimorphic characters, which are the pale staining of the cytoplasm and the large diffuse centrally located nuclei; dumbbell-shaped growing gametocytes (Fig. 2j) are seen occasionally; other characters are as for macrogametocytes.

*Taxonomic remarks:* This is the first species of *Haemoproteus* described in birds of the Prunellidae. It should be distinguished from haemoproteid species described in birds of the closely related families belonging to Passerida (Dicaeidae, Estrildidae, Fringillidae, Motacillidae, Nectariniidae, Passeridae, Ploceidae). Due to the markedly variable position of the nuclei in the macrogametocytes (Fig. 2e–g), the predominantly elongate pigment granules (Fig. 2f), the modest nuclear displacement ratio (Fig. 2f–h; Table 2), *H. bobricklefsi* sp. nov. can be readily distinguished from 16 *Haemoproteus* species, which were described in birds of the Passerida (Valkiūnas and Iezhova, 2022). The new species should be distinguished from the morphologically particularly similar *H. anthi*, *H. dolniki*, *H. fringillae* and *H. motacillae*. Macrogametocyte nuclei are strictly subterminal in position in all these parasites (Valkiūnas, 2005; Valkiūnas and Iezhova, 2022), which is not the case in *H. bobricklefsi* sp. nov. (see Fig. 2e–g).

### Host and geographical distribution

In Europe, the lineage hDUNNO01 was detected and confirmed using microscopic examination only in dunnocks, although it has been detected infrequently using PCR-based diagnostics in some other bird species (Table 1). It is probably a specialist parasite. Gametocytes of this parasite were seen only in dunnocks. Fully grown macrogametocytes with unstable position of nuclei – the characteristic feature of *H. bobricklefsi* sp. nov. (see Fig. 2e–g) – were not seen in European passeriform birds. The available data suggest that prevalence of the hDUNNO01 infection decreases from western (the UK) to eastern (Sweden, Lithuania) areas of this bird distribution in Europe (Table 1).

Prevalence data for the lineage hDUNNO01 in North America are available for birds of the Passerellidae (Table 1); only results of molecular diagnostics are available, but the presence of gametocytes has not been documented in all of them. Transmission certainly occurs in North America; however, the competent vertebrate hosts and vectors remain unidentified.

**Figure 3.**
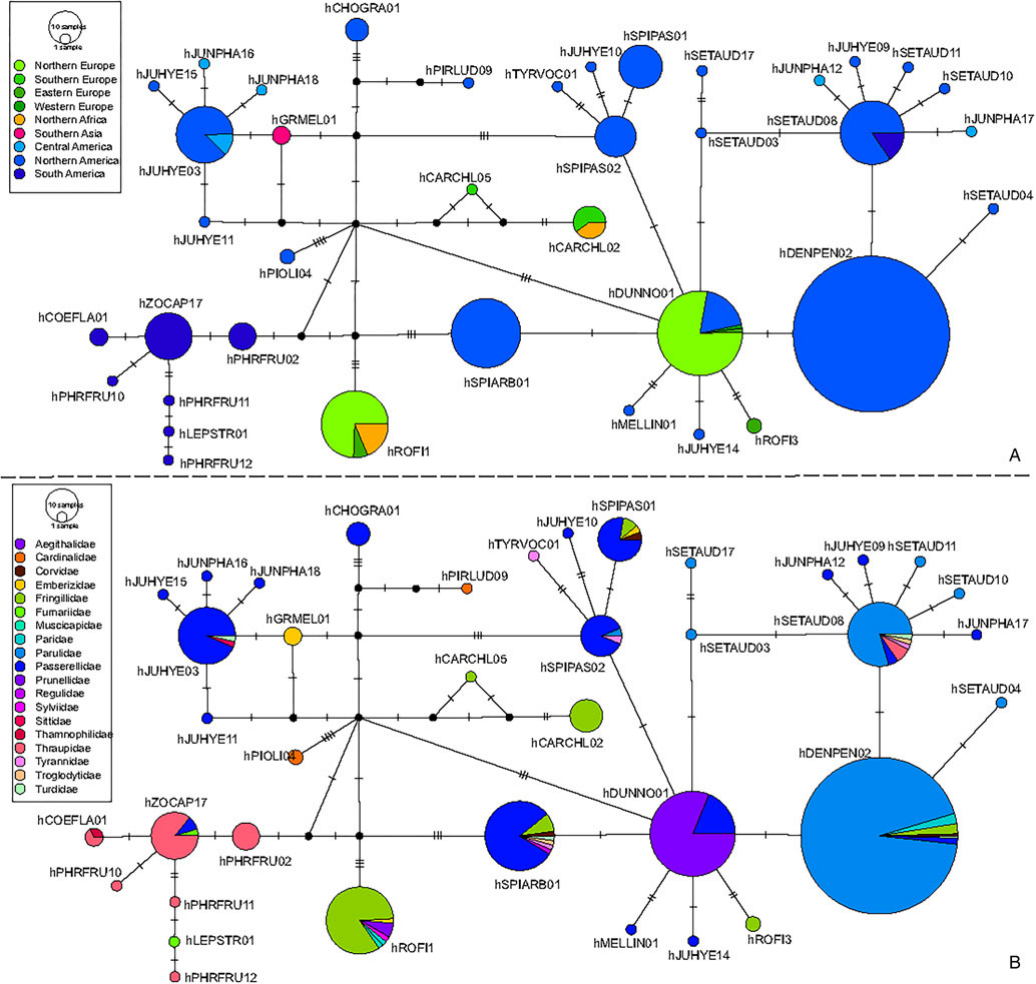
Median-Joining DNA haplotype networks of partial cytochrome *b* gene sequences (478 bp). Lineages were selected to be within 1.5% difference from hDUNNO01, the target lineage of this study. Traits are the geographical areas, according to the United Nations geoscheme, were shown for each positive individual (A) and the host families (B). One parasite lineage corresponds to 1 circle with its frequency related to the size of the circle. Hatch marks represent the number of mutations between 2 lineages. Small black circles represent hypothetical missing links, the lineages needed to connect 2 lineages with the maximum parsimony. The lineage hDUNNO01 was mostly found in Northern Europe in the Prunellidae species and in Northern America in the Passerellidae birds. Most lineages were from the Americas (A); these were present mainly in Passerellidae and Parulidae birds (B). The lineages recorded in Europe (A) were mostly recorded from the Fringillidae birds (B).

A total of 37 lineages were found within a sequence divergence of 1.5% from hDUNNO01 partial *cytb* gene sequence. Four of these lineages were recorded in Europe and mostly in the Fringillidae birds (hCHARCHL02, hCARCHL05, hROFI1 and hROFI3), 1 in Asia in Emberizidae species (hGRMEL01), while all others were from the Americas and predominantly reported in the birds belonging to the Passerellidae and Parulidae (Fig. 3A and B). Lineages recorded in South America (hPHFRU02, hPHFRU10, hPHFRU11, hPHFRU12, hZOCAP17 and hCOEFLA01) were mostly reported in birds of the Thraupidae. The lineage hDUNNO01 was mostly reported in Northern Europe, with the highest number of records in the UK in the dunnock (Prunellidae), and in Northern America in birds of the Passerellidae (Fig. 3A and B; Table 1).

## Discussion

The morphological data and phylogenetic analysis coincided in regard of the validity of *H. bobricklefsi* sp. nov. Mainly, this parasite is readily distinguishable at the gametocyte stage and is distinct in the phylogenetic trees. Interestingly, the phylogenetic analyses based both on the partial *cytb* gene and the full mitochondrial genome showed the same trend of relationships between *H. bobricklefsi* sp. nov. and the closely related species, indicating the value of short *cytb* sequences in estimating of relationships between *Haemoproteus* parasites. Genetic differences between these parasites were also similar when comparing the partial *cytb* gene with the full mitochondrial genome.

It is worth noting that we developed the phylogenetic tree using only *cytb* sequences obtained from the type avian hosts of parasites for the first time (Fig. 1A). Due to the relatively high specificity of *Haemoproteus* pathogens to avian hosts (Atkinson, 2008; Valkiūnas and Atkinson, 2020), such an approach minimizes the incorporation of incorrect parasite species identifications in phylogenetic analyses and makes the evolutionary hypotheses more rational in regard to parasite taxonomy. Unfortunately, incorrect identifications remain in GenBank (Valkiūnas *et al*., 2008) and, accordingly, they might be incorporated in phylogenetic analyses, making the evolutionary considerations questionable simply from the parasite taxonomy perspective. This is particularly recognizable when the same parasite names are scattered over the large phylogenetic trees (Mantilla *et al*., 2016; Lotta *et al*., 2019). Such trees are helpful for distinguishing possible cryptic or morphologically similar parasite species but can be misleading from the point of view of phylogenetic conclusions on relationships among certain parasite species.

According to the available molecular records of the lineage hDUNNO01, *H. bobricklefsi* sp. nov. is of the Holarctic distribution (Table 1, Fig. 3A) and might be restricted in transmission to countries with relatively cool climates. All documented findings of this lineage came from sites located approximately north of 48° latitude in Europe and 39° in the USA (Table 1). Interestingly, most accentor birds breed in relatively cool alpine environments and near the upper timberline in high mountains (Liu *et al*., 2017). *Haemoproteus bobricklefsi* sp. nov. might be adapted for transmission at relatively chilly environmental conditions. Among the accentor birds, *Haemoproteus* parasites have been reported only in the dunnock thus far, but all other *Prunella* species remain non-sampled. Certain areas of *H. bobricklefsi* sp. nov. transmission remain unclear in Eurasia because there are no data from Siberia and other regions of Asia where most species of *Prunella* breed. However, the available molecular records for the lineage hDUNNO01 show that this parasite certainly is transmitted in Europe and North America (Table 1). It has not been reported in other parts of the world thus far.

In total, the lineage hDUNNO01 was found in birds of 17 species, 14 genera, 7 families and 2 orders (Table 1). Most reports were from the Passeriformes birds, except 1 record in Galliformes birds (Oakgrove *et al*., 2014). In Europe, hDUNNO01 was found mostly in species of Prunellidae, but occasionally also in Emberizidae, Turdidae and Phylloscopidae birds (Dunn *et al*., 2014; Woodrow *et al*., 2023; JCD, unpublished data). Gametocytes were seen only in the dunnock, which is the only known competent host of *H. bobricklefsi* sp. nov. thus far. There is a record of the lineage hDUNNO01 from yellowhammer *Emberiza citrinella* in the UK (Dunn *et al*., 2014), but it is unclear if gametocytes develop in this bird species. Eighteen yellowhammers were examined in Ventė Cape, Lithuania but hDUNNO01 was not found (Duc *et al*., 2023); only gametocytes of *H. dumbbellus* were present in this bird species with the prevalence of 44%. Fifty examined yellowhammers were negative for hDUNNO01 at Krankesjön, Sweden (Ellis *et al*., 2020), and 61 yellowhammers were negative for hDUNNO01 elsewhere in the UK (JCD, unpublished data). The lineage hDUNNO01 was also found in 2 of 106 (1.9%) sampled common blackbirds *Turdus merula*, 1/58 (1.7%) Eurasian wrens *Troglodytes troglodytes* and 1/16 (6.3%) willow warblers *Phylloscopus trochilus* (JCD, unpublished data). Interestingly, all these bird species were positive only in the UK where the prevalence of hDUNNO01 infection in dunnocks was high (50%), indicating a possibility of cross-infection of *H. bobricklefsi* sp. nov. between dunnocks and other passerines. Because gametocytes of *H. bobricklefsi* sp. nov. have never been seen in other birds than dunnocks in Europe, all these reports might be cases of abortive infections after inoculation of sporozoites by vectors in non-competent hosts at areas of the active transmission.

The abortive *Haemoproteus* infections might be virulent in non-adapted hosts (Valkiūnas and Iezhova, 2017; Ortiz-Catedral *et al*., 2019). This raises a question about health consequences of the *H. bobricklefsi* sp. nov. development in species other than dunnocks. Bird populations in the UK might be convenient to address this issue due to the high prevalence of *H. bobricklefsi* sp. nov. in dunnocks, indicating active transmission. Collection of organ samples from birds, which are hDUNNO01 lineage positive, can be recommended for histological examination aiming to learn if this parasite produces tissue stages and harms bird species other than dunnocks in the UK. Incidentally dead dunnocks and other birds killed during collisions against constructions such as buildings, bridges, lighthouses and others can be collected and preserved for histological examination. Citizen science can be applied for this purpose as well (Himmel *et al*., 2021).

In North America, the lineage hDUNNO01 was most often reported in Passerellidae species (Table 1, Fig. 3B), noteworthy in the same superfamily Passeroidea to which the family Prunellidae also belongs. A few reports were also from birds of Passeridae, Turdidae and Phasianidae (MalAvi database, accessed February 2024). Because there is no evidence that *Haemoproteus* species are transmitted and produce gametocytes (invasive stages for vector) in birds belonging to different orders (reviewed by Valkiūnas, 2005; Atkinson, 2008; Valkiūnas and Iezhova, 2022), the report in willow ptarmigans *Lagopus lagopus* (Galliformes) shows that abortive infections of this lineage likely occur in wildlife, and some other reports in the American passerine birds might be cases of incomplete (abortive) development. That is not unexpected due to similar abortive-development reports of *Haemoproteus witti* – the specific parasite of Apodiformes birds – in many species of American passerines (Moens *et al*., 2016). Competent hosts of the hDUNNO01 lineage remain unidentified in North America because none of the available studies were accompanied by microscopic examination of blood samples; thus, there is no information as to whether gametocytes develop in these avian hosts.

*Haemoproteus erythrogravidus* (the parasite type host is the rufous-collared sparrow *Zonotrichia capensis*) and *Haemoproteus coatneyi* (the white-throated sparrow *Zonotrichia albicollis*) also parasitize Passerellidae birds in Americas (Mantilla *et al*., 2016). These 2 parasites are phylogenetically closely related to *H. bobricklefsi* sp. nov. (hDUNNO01), but they are readily distinguishable morphologically and genetically in complete mitochondrial genome and the barcoding MalAvi database *cytb* sequence (Fig. 1A and B). This indicates the existence of a relatively diverse group of related *Haemoproteus* species parasitizing birds of Passerellidae. Due to the larger number of bird species positive for hDUNNO01 in North America than in Europe (Table 1), it might be that this parasite originated in North America in Passerellidae species and then colonized Europe. This hypothesis is in agreement with the data that most lineages, which are closely related to hDUNNO01, were found exclusively in the Americas (Fig. 3B). It is interesting that the species *Haemoproteus tartakovskyi* (hSISKIN1, Fig. 1), which is related to *H. bobricklefsi* (hDUNNO01), also has a transmission range including both Europe and North America. Previous analyses of partial *cytb* gene and nearly 1000 nuclear genes suggested that the lineage hSISKIN1 might have originated in North America and subsequently spread to Europe (Huang *et al*., 2019). Due to the close phylogenetic relationships of these lineages and the similar pattern of geographical distribution, we speculate that the same mode of global spread might have occurred during the evolution of *H. bobricklefsi* (hDUNNO01).

The lineage hDUNNO01 appeared in the clade of *Parahaemoproteus* species in the phylogenetic analyses (Fig. 1), indicating that this parasite is likely transmitted by *Culicoides* biting midges, as it is the case with all studied parasite species of the *Parahaemoproteus* clade (Bukauskaitė *et al*., 2019; Chagas *et al*., 2019). Interestingly, this lineage was isolated from salivary gland samples of *Culex pipiens* and *Culiseta annulata* mosquitoes in the UK (JCD, personal observation). Because sporogony of *Haemoproteus* species does not complete in mosquitoes, these reports probably are examples of abortive development in mosquitoes. This raises the question of what the templates for PCR amplification might be in samples from the salivary glands if sporozoites are absent? *Haemoproteus* parasites readily exflagellate and produce ookinetes in mosquitoes, and the ookinetes actively migrate *via* haemocoel all over the body of the exposed insects. Interestingly, positive PCR signals of *Haemoproteus* parasites were revealed in experimentally infected mosquitoes during the abortive development as long as 15–17 days post exposure (Valkiūnas *et al*., 2013), showing a relatively long survival of the arrested parasites in the resistant insects without DNA degeneration. *Haemoproteus* ookinetes were visualized in thoraxes of blood-sucking insects, and they can serve as a source of DNA for PCR amplification (Valkiūnas *et al*., 2013, 2014; Bukauskaitė *et al*., 2016). It is difficult to rule out that the ookinetes can contaminate the salivary gland samples during abortive developments as is the case with the adjacent tissues of the thorax. In other words, even if salivary glands are expected to be useful for molecular detection of haemosporidian parasites, it remains essential to confirm the presence of sporozoites for definitively demonstrating the insects are vectors. This is a particularly sensitive issue in studies aiming for establishing novel groups of haemosporidian parasite vectors. Certainly, PCR-based diagnostics should be combined with microscopic examination during vector studies of haemosporidians because the former approach does not distinguish abortive parasite development.

The dunnock is a common bird species in sites of dense vegetation in the UK, with an overall population estimated at 2.5 million pairs (British Trust for Ornithology, 2023). Targeted epidemiological research is needed for understanding factors contributing to the remarkably high prevalence of *H. bobricklefsi* sp. nov. in dunnocks in the UK in comparison to other studied sites (Table 1). The high prevalence might be related to the high density of the dunnock population, the attractiveness of this bird species to biting midges, and the non-migratory behaviour of the dunnock, resulting in exposure to bites of vectors and active transmission during most of the year in the UK. Biting midges – the likely vectors of this parasite – are active from April to November in the UK (Sanders *et al*., 2011), providing opportunities for a prolonged period of transmission. This is not a case in the Eastern Baltics and Sweden where the dunnock is less abundant and migratory (Logminas *et al*., 1991; Jusys *et al*., 2012; Keller *et al*., 2020), resulting in a shorter period of exposure to vectors and more restricted opportunities for transmission during the breeding period, partially due to a shorter activity season (May – beginning of October) of biting midges (Bernotienė *et al*., 2019).

## Conclusions

This study described a new avian haemosporidian parasite – *H. bobricklefsi* sp. nov. (lineage hDUNNO01) – which is transmitted in Europe and North America. The dunnock is the only known competent host, in which transmissible stages (gametocytes) so far have been documented. This lineage predominantly infects species of the families Prunellidae (in Europe) and Passerellidae (in North America). *Haemoproteus bobricklefsi* sp. nov. has a remarkably patchy distribution in Europe, and it presumably colonized Europe from North America. Molecular diagnostics shows that *H. bobricklefsi* sp. nov. is most prevalent in the UK where the dunnock population is dense and non-migratory. These 2 factors seem important for the active transmission of *H. bobricklefsi* sp. nov. active transmission. Phylogenetic analysis suggests that this haemosporidian parasite belongs to subgenus *Parahaemoproteus* and is likely transmitted by *Culicoides* biting midges. Numerous PCR-based records of the lineage hDUNNO01 in bird species, in which gametocytes of *H. bobricklefsi* sp. nov. have never been observed, suggest that this parasite is prone to abortive development in ‘wrong’ hosts, calling for research aiming better understanding of its exo-erythrocytic development in relation to avian health. The UK – the site of remarkably high prevalence of this infection – is convenient for material sampling to research this issue.

## Data Availability

Data will be available on request.
